# Classifying the difficulty levels of working memory tasks by using pupillary response

**DOI:** 10.7717/peerj.12864

**Published:** 2022-03-29

**Authors:** Hugo Mitre-Hernandez, Jorge Sanchez-Rodriguez, Sergio Nava-Muñoz, Carlos Lara-Alvarez

**Affiliations:** Unidad Zacatecas, Centro de Investigación en Matemáticas, A.C., Zacatecas, Zacatecas, Mexico

**Keywords:** Working memory, Pupil size, Cognitive load, Classifiers

## Abstract

Knowing the difficulty of a given task is crucial for improving the learning outcomes. This paper studies the difficulty level classification of memorization tasks from pupillary response data. Developing a difficulty level classifier from pupil size features is challenging because of the inter-subject variability of pupil responses. Eye-tracking data used in this study was collected while students solved different memorization tasks divided as low-, medium-, and high-level. Statistical analysis shows that values of pupillometric features (as peak dilation, pupil diameter change, and suchlike) differ significantly for different difficulty levels. We used a wrapper method to select the pupillometric features that work the best for the most common classifiers; Support Vector Machine (SVM), Decision Tree (DT), Linear Discriminant Analysis (LDA), and Random Forest (RF). Despite the statistical difference, experiments showed that a random forest classifier trained with five features obtained the best F1-score (82%). This result is essential because it describes a method to evaluate the cognitive load of a subject performing a task using only pupil size features.

## Introduction

Several authors try to explain how memory works. The well-known multi-store model (Atkinson & Shiffrin, 1968) compares the structure of the human brain with a computer. Atkinson & Shiffrin (1968) consider that the information flows between three cognitive structures. First, it is acquired from sensory registers that perceive sights, sounds, *etc.* When we put attention to these stimuli, the information moves to the short-term (STM), a.k.a. working memory. The STM is a cognitive system with a limited capacity used to remember information over a brief time. Deep processing transfers the STM information into the more persistent and virtually unlimited storage called long-term memory(LTM). James (2007) assumes a dichotomous memory model, consisting of primary memory and secondary memory. The information in the primary memory lasts for a few seconds and holds information in our consciousness. The information in the secondary memory has unlimited duration and can be brought to consciousness if desired.

The multi-store and the dichotomous models cannot explain case studies where the verbal STM is impaired while the visual STM is normal. Baddeley & Hitch (1974) propose an initial multi-component model that explains this phenomenon. This model considers that the working memory (WM) has at least three interacting systems: the central executive system, the visuo-spatial sketch-pad, and the phonological loop. In this model, the STM refers to simple temporary storage of information, in contrast to WM, which implies a combination of storage and manipulation (Baddeley, 2012). The central executive system controls cognitive processes by coordinating two slave systems: the visuo-spatial sketch-pad and the phonological loop. These subsystems can store verbal and visuo-spatial data, respectively. In other work, Baddeley (2000) included another component, the episodic buffer. This subsystem can integrate visual, spatial, and verbal information with time sequencing. Furthermore, this component links Working Memory to perception and Long-Term Memory. The episodic buffer is assumed to have a limited capacity of about four chunks or episodes, and to be accessible through conscious awareness (Baddeley, 2010).

Cognitive load can be defined as the load that performing a task imposes on the learner’s cognitive system (Paas & Van Merriënboer, 1994). Cognitive load theory suggests that the learning content must be designed in such a way that it does not exceed the processing capacity of the learner (Sweller, 1988; Mayer & Moreno, 2003). Tasks such as language comprehension, learning, and reasoning require organizing and processing information in the working memory (Baddeley, 1992). As the working memory has a limited capacity, instructional methods should avoid additional activities that do not directly contribute to learning. Then, it is necessary to evaluate the cognitive load caused by a given task.

While previous studies have shown the pupil size differences for different task difficulties, this research aims to build a multi-class classifier that can identify the difficulty level of a memorization task by analyzing eye tracking data.

For this aim, we use the pupil size features from individuals solving the well-known *Digit Span* test. The digit span consists of evaluating the ability of an individual to remember a sequence of numbers. Some tools (*e.g.*, surveys) can be used to predict the cognitive load caused by a task in a specific group. The type of task and the subject’s characteristics (capabilities, cognitive style, preferences, and previous knowledge) are important factors that influence the cognitive load (Paas & Van Merriënboer, 1994). Moreover, several factors affect working memory; *e.g.*, anxiety (Klados et al., 2015).

This work hypothesizes that we can determine the task difficulty perceived by an individual from her pupil-size features such as the mean pupil diameter change, the average percentage change in pupil size, peak dilation, and time to peak. If such a hypothesis holds, then a low-cost oculometer can be used massively to adjust the difficulty of a task in real-time. Knowing the difficulty of a given task is crucial for improving the learning outcomes. A question that arises is could a limited sensor give enough information for measuring the difficulty of a task in real-time? Physiological or behavioral data has the advantage of being available in real-time. Examples of physiological data are pupil responses, heart rate, blood glucose, blood pressure, respiration rate, body temperature, *etc*. Besides, behavioral data refer to how people interact with objects or other people. Developing a difficulty level classifier from pupil size features is challenging because of the inter-subject variability of pupil responses. Eye-tracking data used in this study was collected while students solved different memorization tasks divided as low-, medium-, and high-level.

The research questions addressed by this study are: (i) Are there significant differences in the study variables (features) due to the difficulty level? (ii) Can we obtain a good classification performance of the difficulty level of a memorization task by feeding pupil size features into popular classifiers? (iii) What is the best configuration (classifier/features)?

The multi-component model (Baddeley, 2010) exposes that the visuo-spatial sketch-pad and the phonological loop components have a limited capacity and that are relatively independent of each other. Klingner, Tversky & Hanrahan (2011) studied the effects in pupil dilation evoked by a digit-span memory task presented aurally and visually. They found that aural presentation caused significantly larger pupil dilations during the retention pause (M 0.44 mm, SD 0.22 mm) than visual presentation (M 0.24 mm, SD 0.17 mm). We selected the visual mode data in our experiments because it is more challenging for classification. Experimental results show that features based on pupil dilation can describe the difficulty of memorization tasks, even for visual presentation.

Besides the classical features based on pupil size, which have been used in numerous classification approaches, we also essay two new features: the *peak dilation speed* and the *entropy of pupil size*. This contribution is valuable for cognitive researchers because one can infer the cognitive load of a task from pupil size metrics. Several studies have shown a significant change in pupil size for harder tasks. For reference, we evaluate the differences in pupillary responses for three difficulty levels of the digit span task. In general, features showed statistically significant differences for different difficulty levels. The only exception to this was a lack of statistically significant differences in the baseline. However, results also show that the best classifier also considers this variable.

### Related work

As the earlier work showed, pupil size is an indicator of mental activity. Hess & Polt (1964) observed that the pupil response is closely correlated with the difficulty of an arithmetical problem. Kahneman & Beatty (1966) was the first work that explores the influence of cognitive load on pupil diameter. They asked participants of an experiment to remember a sequence of digits and nouns. They observed that the pupil dilates as the material is presented and constricts during report.

The cognitive load is usually measured by (i) *surveys* such as the NASA-TLX (Hart, 2006) or the instructional approach (Eysink et al., 2009); (ii) *physiological measures* such as electroencephalogram (Lin & Kao, 2018), Galvanic Skin Response (Nourbakhsh et al., 2017), or eye size (Marandi et al., 2018); or (iii) *behavioral data* as the eye movements (Marandi et al., 2018). However, surveys do not offer real-time information, and physiological skin contact devices can be uncomfortable. An option is to use a remote eye-tracker. This is ideal for long-term use (*i.e.,* continuous use for more than 30 days) because it is usually placed under the screen at 50–70 centimeters from the user.

Common eye-tracker information used to detect cognitive load are the eye-pupil size (Kun et al., 2013; Palinko & Kun, 2012), saccades (Marandi et al., 2018), fixations (Nocera, Camilli & Terenzi, 2006), and blinks (Faure, Lobjois & Benguigui, 2016). Fixations and saccades are made up of multiple gaze points. Examples of gaze features used to describe cognitive load are the length (Mitre-Hernandez et al., 2019), peak velocity (Di Stasi et al., 2010), and movements of saccades (Marandi et al., 2018). Eye blinking is influenced by cognitive processes. In general, the blink frequency drops when the task difficulty increases (Marandi et al., 2018; Faure, Lobjois & Benguigui, 2016). The blink frequency also is affected by changes in auditory tasks; *e.g.*, questions for planned or spontaneous answers (Mitre-Hernandez et al., 2019), interviews (Frosina et al., 2018), or yes/no questions (George et al., 2017). From a statistical analysis of data generated from a driving task, Kun et al. (2013); Palinko & Kun (2012) observe a statistical difference in the pupil size for conversational tasks that increase the cognitive load and those that decrease it. Here, we analyze the statistical significance of different pupil-size indicators and the difficulty of a task. Furthermore, we use these pupil-size indicators as features in several classifiers that predict the difficulty of a memorization task.

Intuitively, the more information we use, the better performance we reach. That is why supervised learning approaches that use eye-tracker data (Table 1) use several features. But, in real applications, researchers face situations where some features cannot be used (*e.g.*, in a language conversation practice, fixations or saccades cannot be available). Besides, some features can be voluntary controlled by the subject. Physiological signals reflect unconscious body changes, and are controlled by the sympathetic nervous system, while visual and audio cues can be adopted voluntarily or involuntarily (Shu & Wang, 2017).

**Table 1:** Supervised learning approaches that used eye-tracker data. Bold numbers indicate the best accuracy.

**Author**	**Classes**	**Features**	**Classifier**	**Accuracy**
Biedert et al. (2012)	Reading, skimming	fixations features	SVM (RBF)	80.0
		saccades features		79.0
		fixations and saccades features		**88.9**
Kollmorgen & Holmqvist (2007)	Reading, no reading	fixation time, saccade size	HMM	**91.0**
			HMM (online)	88.0
Kunze et al. (2013)	Type of reading: novel, manga, journal, newspaper, book	fixation and saccade features	Decision tree	75.0
Eivazi & Bednarik (2011)	cognitive patterns: cognition, evaluation, planning, intention	fixation features	SVM (RBF)	53.3
	Performance: low, medium, high	mean time of actions	SVM (RBF)	66.5
Li et al. (2020)	Mental fatigue: low, medium, high	21 pupil size and blinks features	SVM	85.0
			Decision tree	78.4
			Boosted tree	81.0
			KNN	76.5
			LDA	**86.0**
		56 blinks and gaze features	SVM	**81.3**
			Decision tree	79.4
			Boosted tree	71.5
			KNN	63.4
			LDA	81.2
		63 gaze features	SVM	79.5
			Decision tree	79.7
			Boosted tree	73.6
			KNN	63.9
		LDA	**80.5**
		70 gaze and eye-fixation	SVM	84.1
			Decision tree	**87.1**
			Boosted tree	75.4
			KNN	73.9
			LDA	78.8
Deng et al. (2019)	Driving behavior: change to the left lane, right, and keep in the lane	E = 51 saccades, blinks, and gaze features	SVM	70.32
			HMM	64.94
			CNN	86.19
			RF	**93.66**
		V = {vehicle speed, distance to the front, front-left, right-left, and back vehicles, time to collision, lane number}	SVM	92.45
			HMM	93.66
			CNN	89.25
			RF	**99.14**
		*E*∪*V*	SVM	79.79
			HMM	94.37
			CNN	90.35
			RF	**99.92**

Our ultimate goal is to develop a real-time method that can classify the perceived difficulty of different tasks (as olfactory, verbal, or visual ones) from physiological eye measurements. These considerations reduce the set of possible eye features just to pupil size and blink features. Unfortunately, eye blinking is only noticeable for frequency measurements of at least 100 Hz (Nakamura et al., 2008). That is why this paper focuses on pupil size features for difficulty classification.

Classification is the problem of identifying the category of a given observation. Classification from ocular features has been used in diverse applications such as detecting types of reading (Biedert et al., 2012; Kunze et al., 2013), mental states (Eivazi & Bednarik, 2011), levels of mental fatigue (Li et al., 2020), and driving behaviours (Deng et al., 2019). Another difference between the different approaches shown in Table 1 is the classifier selection. In the following paragraphs, we overview the related work concerning the classifier used.

A Support-Vector Machine (SVM) estimates the hyperplane that separates two classes with the maximal margin (Cortes & Vapnik, 1995). The original SVM only solves binary classification problems. Many techniques extended it to multiclass classification, the general idea is to predict the class of an instance form several binary SVM classifiers. As it is shown in Table 1, the SVM gives acceptable results for predicting mental fatigue (Li et al., 2020); but inconsistent results for predicting driving behaviour (Deng et al., 2019). The SVMs can also manage non-linear classification problems by incorporating a kernel function that maps the inputs into high-dimensional feature spaces. The radial basis function kernel (RBF) is popular to solve many classification problems.

The accuracy of the SVM (RBF) is good for binary classification—*e.g.*, (Biedert et al., 2012); but, as expected, the accuracy is lower for multiclass problems—*e.g.*, (Eivazi & Bednarik, 2011).

Fisher’s linear discriminant (FLD) is in essence a technique for dimension reduction (Fisher, 1936). For two classes, the FLD selects a projection that maximizes the class separation. FLD extension to multiclass is known as linear discriminant analysis (LDA). Its objective is to find a dimension reducing transformation that minimizes the scatter within each class and maximizes the scatter between classes in a reduced dimensional space Kim, Drake & Park (2007). Experiments of Li et al. (2020) to classify mental fatigue show that LDA gives the best results for some features.

The Hidden Markov Model (HMM) considers that a set of observable variables are generated by a sequence of internal (hidden) states. Besides, future hidden states depend only on the present hidden state (Baum & Petrie, 1966). HMM can be used for supervised learning by estimating its parameters from a set of labeled sequences. In this sense, HMM has been used to analyze labeled sequences of saccades, blinks, and other gaze features (*e.g.*, fixation time). For instance, Kollmorgen & Holmqvist (2007) use HMM to detect reading and Li et al. (2020) classify driving behavior.

Decision trees are simple classifiers where a set of decision nodes are arranged in a tree structure. This approach learns rules that best split the learning dataset. Decision threes show average performance for detecting the type of reading (Kunze et al., 2013) and mental fatigue (Li et al., 2020). Boosted trees and Random forests (RF) are classifiers based on decision trees (Ho, 1998). Boosting is a method of combining many weak learners(trees) into a strong classifier. RF uses a feature bagging (bootstrap aggregating) procedure; *i.e.,* it integrates the prediction of several decision trees trained by a random subset of features. Decision and boosting trees obtain inconsistent results for classifying mental fatigue. On the contrary, the RF obtains the best accuracy for discriminating the driving behavior.

In this paper, we study different classifiers to select the best one. Also, we use a feature selection approach to discover the best configuration.

## Materials & Methods

### Dataset

Short-term recall of a packed sequence of digits is a common experimental task in cognitive pupillometry. In this task, participants see (or hear) a sequence of numbers; then, they were asked to recall the sequence correctly, with increasingly longer sequences being tested in each trial. We use the dataset proposed in Klingner, Tversky & Hanrahan (2011) which evaluates different effects for cognitive tasks. In particular, we use pupil dilation measurements for the digit sequence recall task. Sections ‘participants’ and ‘materials’ describe the experiment performed by Klingner, Tversky & Hanrahan (2011).

#### Participants

Twenty-four Stanford undergraduates with normal vision participated in the experiment of Klingner, Tversky & Hanrahan (2011), excluding those with contact lenses or eyeglasses–presenting astigmatism or refractive correction greater than 10 diopters. The authors introduced a monetary incentive for participants, $15 for participants with the lowest scores, and about $35 for the highest.

#### Materials

The visual response of participants was recorded with a remote eye-tracking device Tobii 1750 at 50 Hz. The task was presented on a standard LCD computer display (1, 280 × 1, 024 screen resolution, 17 inches on the diagonal, w:h ratio of 5:4). Experiments were recorded with infrared lights and a high-resolution infrared camera. The eye-tracker supports head movement, but for eye pupil data, the speed movement must be less than 10 cm/s within a head-box of 30 × 15 × 20 cm with an initial distance of 60 cm from the screen. To avoid missing ocular data when participants interact with the screen, the dataset authors placed the eye-tracker on the top of the screen. The experiment room was relatively bright, with 27 cd/m2 of luminance from the surrounding walls at eye level and 32 lx incident at participants’ eyes.

**Figure 1:**
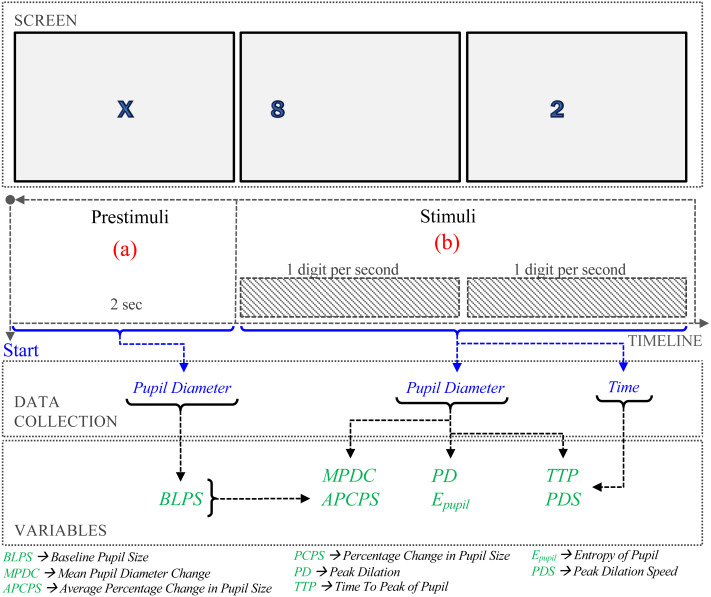
Procedure of the experiment.

### Variables

The following variables were calculated from the pupillary data:

**Baseline Pupil Size (BLPS).** The prestimulus phase lasts two seconds before the question, Fig. 1(a). The BLPS is used to set a value of the pupil stabilization. In our experiments, this value is obtained as the average pupil diameter in the prestimulus phase.

**Mean Pupil Diameter Change (MPDC).** (Palinko et al., 2010; Palinko & Kun, 2012; Kun et al., 2013). To estimate the MPDC, the baseline is subtracted from the average of the pupillary data, that is,

(1) \begin{eqnarray*}\text{MPDC}= \frac{1}{N} \sum _{i=1}^{N}{\text{PS}}_{i}-\text{BLPS},\end{eqnarray*}

where PS_*i*_ is the pupillary data collected at time *i*, and BLPS is the baseline.

**Average Percentage Change in Pupil Size (APCPS).** (Iqbal, Zheng & Bailey, 2004; Iqbal et al., 2005; Lallé et al., 2015). The *Percentage Change in Pupil Size* (PCPS) is calculated as the difference between the measured pupil size and the baseline pupil size, divided by the baseline pupil size.

(2) \begin{eqnarray*}{\text{PCPS}}_{i}= \frac{{\text{PS}}_{i}-\text{BLPS}}{\text{BLPS}} \end{eqnarray*}

where PS_*i*_ is the pupil size collected in the *i*–th time, and BLPS is the baseline of the pupil size. The APCPS is the average in the measurement interval time.

(3) \begin{eqnarray*}\text{APCPS}= \frac{1}{n} \sum _{i=1}^{n}{\text{PCPS}}_{i}\end{eqnarray*}

where *n* is the number of measurements in the interval.

**Peak Dilation (PD).** The Peak Pupil Dilation (PPD) is defined as,

(4) \begin{eqnarray*}\text{PPD}=\max \nolimits \{ {\text{PS}}_{1},{\text{PS}}_{2},\ldots ,{\text{PS}}_{n}\} .\end{eqnarray*}

To reduce the error caused by different sizes of the human eye, we modified the equation proposed in Marandi et al. (2018) by including the BLPS.

(5) \begin{eqnarray*}\text{PD}=\text{PPD}-\text{BLPS}.\end{eqnarray*}

**Entropy of Pupil (*E*_pupil_)**. Suppose that the pupil dilation is a random variable *S* with possible values *s*_1_, *s*_2_, …, *s*_*m*_ such that *s*_1_ = min{PS_1_, PS_2_, …, PS_*n*_}, *s*_*m*_ = PPD. Consider that *p*_*i*_ = *f*(*i*), where *f*(*i*) is the relative frequency associated with the i-th value *s*_*i*_ (*i.e.,* how often the value *s*_*i*_ happens divided by the number of observations *n*). The information entropy is defined as

(6) \begin{eqnarray*}{E}_{\text{pupil}}=-\sum _{i}^{}{p}_{i}\log \nolimits {p}_{i}\end{eqnarray*}

Entropy can be described qualitatively as a measure of energy dispersal. The concept is linked to disorder: entropy is a measure of disorder, and nature tends toward maximum entropy for any isolated system.

**Time to Peak (TTP).** Siegle, Ichikawa & Steinhauer (2008) show that pupil dilation peaked between the completion of the memory encoding interval time and the start of memory storage. The dilation is proportional to the difficulty of the memory task, as higher the difficulty, higher the peak is. Then, the *time to peak* may reveal the difficulty level. It is defined as,

(7) \begin{eqnarray*}\text{TTP}=\mathrm{time}(\text{PPD}).\end{eqnarray*}

** Peak Dilation Speed (PDS)** The method of least squares can find a relation between time and peak dilation (Siegle, Ichikawa & Steinhauer, 2008). Consider the PS slope of the line that ends at the pupil dilation peak, estimated as

(8) \begin{eqnarray*}m= \frac{\sum {t}_{i}\cdot {\text{PS}}_{i}- \frac{1}{n} \sum {t}_{i}\sum {\text{PS}}_{i}}{\sum t_{i}^{2}- \frac{1}{n} \sum ({t}_{i})^{2}} \end{eqnarray*}

where 0 ≤ *i* ≤ *n*, and *t*_*i*_ and PS_*i*_ are the time and size of the *i*th measurement, respectively. Finally, the slope is used to calculate the angle,

(9) \begin{eqnarray*}\text{PDS}={\tan \nolimits }^{-1}(m).\end{eqnarray*}

### Procedure

As shown in Fig. 1, the procedure consisted of two phases. During pre-stimuli interval time (Fig. 1A), participants observed an ‘X’ in the center of the screen for 2 s. In the stimuli phase (Fig. 1B), digit-span memory tasks were presented randomly. In each task, one digit appears on the screen each second. Based on the number of digits presented to the participant. The cognitive load evoked by memorizing a given number of digits is not linear (Crannell & Parrish, 1957); here, we consider three difficulty levels: the low-level difficulty was associated with memorizing three-digit numbers, medium-level to five-digit numbers, and high-level to eight-digit numbers.

**Figure 2:**
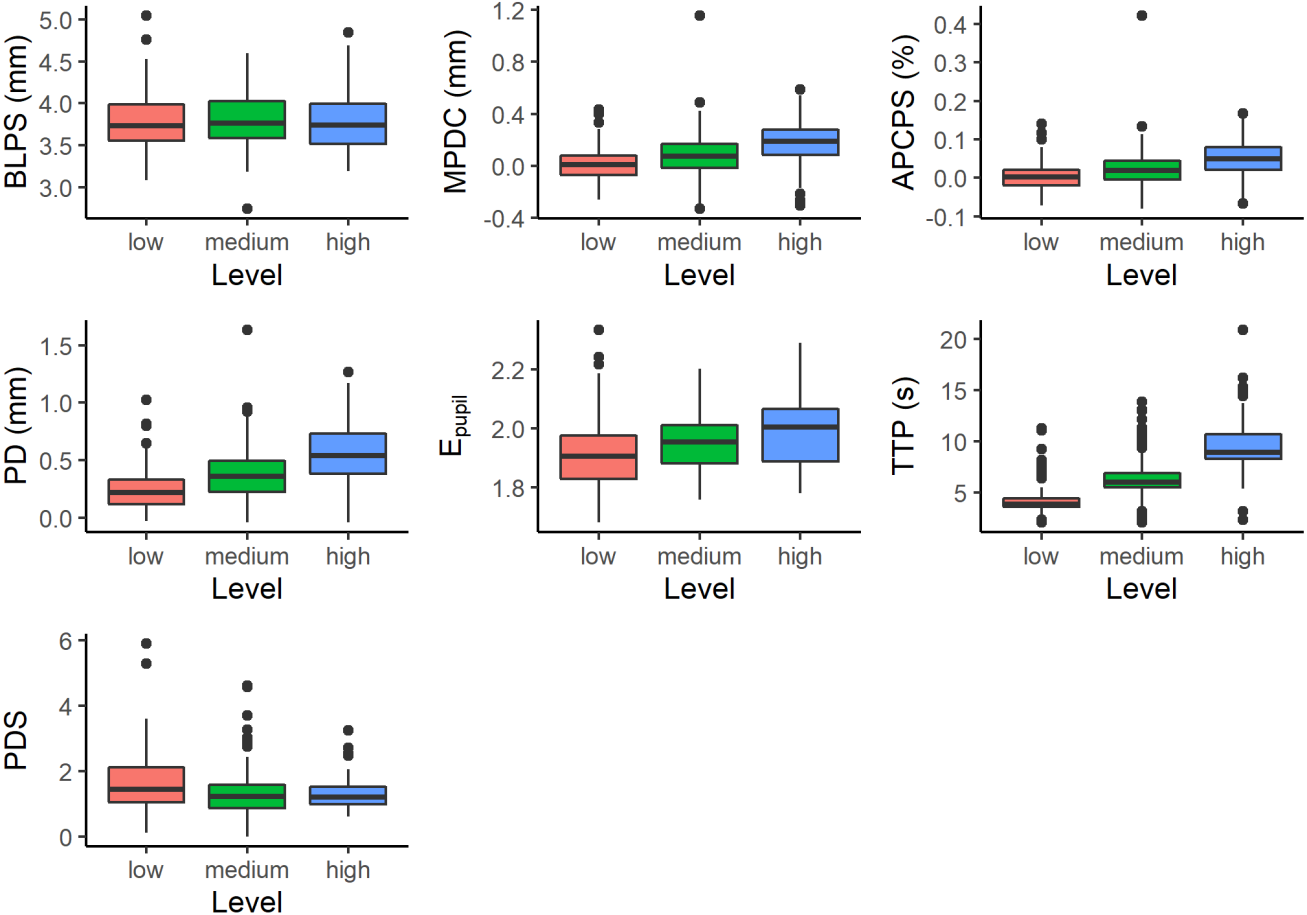
Measurements with respect to difficulty level; Baseline Pupil Size (BLPS), Mean Pupil Diameter Change (MPDC), Average Percentage Change in Pupil Size (APCPS), Peak Dilation (PD), Entropy of Pupil (*E*_pupil_), Time to Peak (TTP), and Peak Dilation Speed (PDS).

The number of pupil size measurements depends on the number of digits presented on the screen. For this reason, a different number of measurements were obtained for each subject at each difficulty level. All of them have at least three measurements. And a maximum of 7, 10, and 13 measurements for low, medium, and high difficulty levels, respectively. Figure 2 shows the measurements for different difficulty levels.

The pupil diameter collected in the prestimuli phase was used for BLPS calculation, and the time and pupil diameter in stimuli phase were used to calculate MPDC, APCPS, PCPS, P_pupil_, PD, TTP, PDS calculation. Finally, each trial was labeled with one of 3 levels of task difficulty (low, medium, and high).

### Data filtering and preprocessing

Data were pre-processed as follows, records with null values or with blinks were eliminated, records with information in both eyes were averaged, and records with data in only one eye were not changed.

A valid trial must not have more than 20% of Invalid records. Finally, the measurements were passed through a Savitzky–Golay filter (Savitzky & Golay, 1964) for the purpose of smoothing the pupil size signal. We choose this filter to preserve characteristics of the initial distribution, which normally disappear with other techniques.

### Statistical analysis

To investigate if there were significant differences in the study variables due to the difficulty level we used a linear mixed model (Demidenko, 1987).

**Figure 3:**
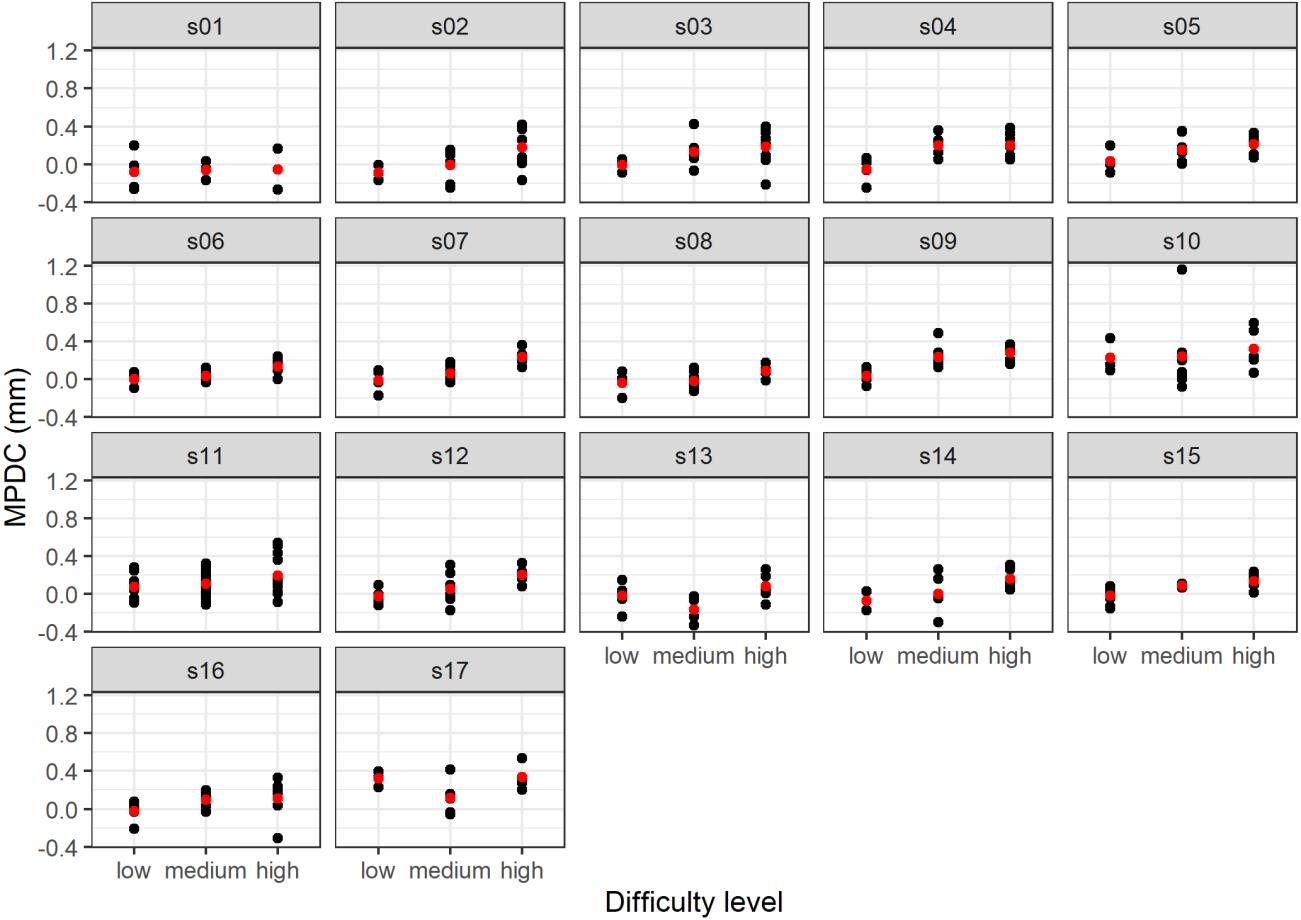
Results of the MPDC measurement *versus* difficulty level by subject (the mean value is shown in red).

Several measurements were taken for each individual at each difficulty level. This process violates the assumption of independence of a linear model. On the other hand, the difficulty results of levels between-subject can present different mean pupil diameter change (Fig. 3).

This characteristic factor affects all the responses of the same subject, which makes these responses interdependent instead of independent. Then, for the analysis, we considered the difficulty level a fixed effect, and both the subject and the subject-level effects random effects.

For the seven variables (BLPS, MPDC, APCPS, E_pupil_, PD, TTP, and PDS) a linear mixed model was adjusted. In particular, the model was estimated using the *restricted maximum likelihood* (REML) approach (Harville, 1977; Patterson & Thompson, 1971). Statistical analysis has been performed utilizing R.

### Classification

After the initial statistical analysis, we selected the most precise five classifiers from those shown in Table 1. The tested classifiers were the SVM with four kernels, LDA, Decision Trees, and Random Forest. The code was implemented in python using the scikit-learn library (version 0.14).

We studied the best classifier configuration. For this aim, subsets of the features set {BLPS, MPDC, APCPS, PD, E_pupil_, TTP, PDS} were tested. With a set of *b* features, 2^*b*^ − 1 different sets can be realized. In this case, with seven training features, we have a total of 127 possible combinations. Each classifier/subset feature pair was trained with 80% of the data and tested with the remaining 20% data. For this aim, we adopted a *k*-fold cross-validation scheme (*k* = 5). In *k*-fold cross-validation, the original sample is randomly partitioned into *k* equal sized subsamples. One of these subsamples was used for testing, and the remaining *k* − 1 subsamples are used as training data.

## Results

### Statistical analysis

We obtained *p*-values by likelihood ratio test of the full model with the effect against the model without the effect in question. As shown in Table 2, we observed that all effects were significant at a level of 0.001, except BLPS which was not significant. After fitting a suitable model, post hoc comparisons were made by using the Tukey HSD test. As shown in Table 3 (‘Initial model’ column), the differences were significant at a level of *p* < 0.05.

**Table 2:** Likelihood ratio tests for different Features: Baseline Pupil Size (BLPS), Mean Pupil Diameter Change (MPDC), Average Percentage Change in Pupil Size (APCPS), Peak Dilation (PD), Entropy of Pupil (*E*_pupil_), Time to Peak (TTP), and Peak Dilation Speed (PDS).

feature	*χ*^2^(2)	*p* − *value*
BLPS	0.01	*p* = 1
MPDC	32.6	*p* < .001
APCPS	29.0	*p* < .001
PD	30.6	*p* < .001
E_pupil_	25.9	*p* < .001
TTP	46.5	*p* < .001
PDS	23.2	*p* < .001

**Table 3:** Tukey HSD Test for different features. The E_pupil_ variable was not transformed because the assumptions were already fulfilled for the original data. Symbols indicate significant differences at levels of †*p* < .1, * *p* < .05, ** *p* < .1, and *** *p* < .001.

Feature	Null hypothesis	Initial model			Final model (without ouliers)		
		Estimate		Std. Error	Estimate		Std. Error
BLPS	medium-low=0	−0.0002		0.031	−0.0053		0.028
	high-low=0	−0.0019		0.028	−0.0154		0.025
	high-medium=0	−0.0017		0.027	−0.0102		0.025
MPDC	medium-low=0	0.061	**	0.023	0.054	*	0.025
	high-low=0	0.167	***	0.023	0.166	***	0.022
	high-medium=0	0.105	***	0.020	0.112	***	0.020
APCPS	medium-low=0	0.017	*	0.006	0.014	†	0.007
	high-low=0	0.044	***	0.007	0.044	***	0.006
	high-medium=0	0.027	***	0.006	0.030	***	0.005
PD	medium-low=0	0.108	***	0.030	0.157	***	0.044
	high-low=0	0.294	***	0.032	0.427	***	0.041
	high-medium=0	0.186	***	0.027	0.270	***	0.033
E_pupil_	medium-low=0	–		–	0.025	**	0.008
	high-low=0	–		–	0.062	***	0.009
	high-medium=0	–		–	0.037	***	0.007
TTP	medium-low=0	2.105	***	0.380	2.117	***	0.372
	high-low=0	5.264	***	0.400	5.178	***	0.363
	high-medium=0	3.159	***	0.314	3.061	***	0.311
PDS	medium-low=0	−0.378	**	0.115	−0.259	*	0.097
	high-low=0	−0.442	**	0.124	−0.243	*	0.095
	high-medium=0	−0.065		0.113	0.016		0.075

A single influential observation (outlier) was found for MPDC, and four influential observations were found for APCPS, TTP, and PDS. Besides, heteroscedasticity of the model residuals for PD and PDS was also observed. Each model was fit to the filtered and normalized data. Table 3 (column ‘final model’) shows the results of the Tukey-HSD post hoc tests of the final models. In general, the conclusions are maintained.

For most variables, the distributional assumptions of the mixed model were not fulfilled. The normality of residuals assumption is the least important one. The linear models are relatively robust against violations of the assumptions of normality. Gelman & Hill (2006), do not even recommend diagnostics of the normality assumption. Indeed, results described in this section show that feature values differ significantly for different difficulty levels.

### Classification of cognitive load

Table 4 shows the accuracy results of the ten best-performing classifiers. The model *M*_1_, a Random Forest classifier trained with five features (BLPS, MPDC, APCPS, PD, and TTP), achieved the best average F1 score. This configuration achieved 85% precision for the low difficulty level, 80% for the medium level, and 83% precision for the low level.

**Table 4:** The best ten out of 127 classifiers results found after the feature selection.

Model			Precision			Average		
Id.	Classifier	Selected Features	low	med	high	prec.	recall	F1
*M*_1_	Random Forest	{BLPS, MPDC, APCPS, PD, TTP}	0.85	0.80	0.83	0.82	0.81	0.82
*M*_2_	SVM (RBF)	{E_pupil_, TTP, PDS}	0.91	0.83	0.67	0.80	0.76	0.77
*M*_3_	SVM (RBF)	{PD, E_pupil_, TTP, PDS}	0.91	0.79	0.69	0.80	0.76	0.77
*M*_4_	SVM (RBF)	{BLPS, APCPS, PS, E_pupil_, TTP}	0.91	0.79	0.69	0.80	0.76	0.77
*M*_5_	SVM (linear)	{MPDC, TTP}	0.83	0.82	0.70	0.79	0.76	0.77
*M*_6_	SVM (sigmoid)	{MPDC, TTP}	0.86	0.83	0.62	0.77	0.75	0.75
*M*_7_	Linear SVC	{BLPS, MPDC, TTP}	0.80	0.72	0.69	0.74	0.70	0.71
*M*_8_	Decision Tree	{APCPS, PD, TTP, PDS}	0.67	0.76	0.64	0.69	0.69	0.69
*M*_9_	LDA	{BLPS, MPDC, APCPS, PD, TTP, PDS}	0.78	0.62	0.75	0.72	0.66	0.67
*M*_10_	LDA	{MPDC, APCPS, PD, E_pupil_, TTP, PDS}	0.78	0.62	0.75	0.72	0.66	0.67

Models based on the Support vector machine (SVM) also achieve good results, with F1 scores as high as 76%. Particularly, the *M*_5_ model uses just two features (MPDC, and TTP), and it is able to reproduce the same F1 score (71%) that other SVM models that use more features.

## Discussion

The relationship between pupil size and mental activity has been studied for a long time (Hess & Polt, 1964). By studying the memory span (the longest list of items that a person can repeat back), Kahneman & Beatty (1966) observe a pupil change of 0.5 mm when going from 3 to 7 memorized digits. Pupil change also occurs in long-term memory retrieval tasks (Papesh, Goldinger & Hout, 2012). Pupil change is evident for both visual and auditory stimuli. Hence, its measurement has found application in different tasks such as: driving a vehicle while listening to a dialogue (Kun et al., 2013), interacting with interfaces for decision making (Lallé et al., 2015), doing math exercises (Beatty, 1982), memorizing numbers from visual stimuli (Beatty, 1982), or performing mental arithmetic operations (Chen, Epps & Chen, 2011). For auditory tasks, pupil dilation presented a larger diameter for hard true/false questions (Webb et al., 2009), interviews (Nugroho, Nasrun & Setianingsih, 2017), and multiple choices (Nurçin et al., 2017). In the Klingner, Tversky & Hanrahan’ experiments, the time to reach maximum dilation from the baseline is in the range of 2,033 to 209,12 ms (*M* = 7, 098 ms). It implies that a low-frequency sensor is good enough to analyze pupil size, which is attractive for many applications. To estimate the pupil change, the experimenter contrasts the size of the pupil at a given moment of the test with a reference size (known as *baseline pupil size*, BLPS). In the conventional experimental procedure, the BLPS is calculated from a stabilization stage that precedes the execution task (Klingner, Tversky & Hanrahan, 2011). Several features can be used; *e.g.*, *peak dilation* (the largest pupil size in the test compared to the BLPS), *time to peak* (the elapsed time from the test beginning until the largest pupil diameter occurred), along with others. The results presented in Table 3 show that there is a strong relationship between pupil size features and the task difficulty. As expected, the baseline pupil size did not show significant differences among different difficulty levels, as the stabilization stage precedes from the task activity.

We evaluated the performance of five classifiers with different combinations of pupil size features. Feature selection is to select a subset of variables from the input that can efficiently describe the input data while reducing effects from noise or irrelevant variables and still provides good prediction results (Guyon & Elisseeff, 2003). Feature ranking methods can use a statistic to select relevant features. Following this approach, TTP could be selected in the first step because it has the best chi-square value (Table 2). Besides, TTP has a significant difference for every 2-combination of the difficulty levels. As shown in Table 4, the TTP feature is present in all the selected features subsets of the best ten classifiers. Wrapper methods use the predictor as a black box and the predictor performance as the objective function to evaluate the variable subset (Chandrashekar & Sahin, 2014). Since evaluating all the subsets becomes an NP-hard problem, suboptimal, subsets are commonly found by employing search algorithms. The set of features in the case study is small (only seven features), then we explored every possible subset. In general, a classifier that uses a combination of features outperforms the use of a single feature, *e.g.*, using TTP alone. For instance, the best model, *M*_1_, is a random forest classifier that uses five out of seven features. The *M*_1_ model obtained an F1 score of 82%. This result is good compared with similar approaches, as in the case of (Eivazi & Bednarik, 2011).

Besides features proposed in the literature, this paper proposes the *peak dilation speed*. This feature is present in five of the best models *M*_2_, *M*_3_, *M*_8_ − *M*_10_. Even though this metric is aligned with empirical findings (Siegle, Ichikawa & Steinhauer, 2008), this feature is not present in the best model. This result could be explained by the relationship between BLPS and the pupil change and the time to peak.

Kahneman (1973) considers that a useful physiological measure of mental effort must be sensitive to within-task, between-tasks, and between-subject variations. That is, such a physiological measure could serve to (i) detect transient variations of the subject’s effort during the performance of a particular task, (ii) order tasks by their difficulty, and (iii) reflect differences in the amount of effort that different people invest in a given task. Beatty (1982) reviews evidence on measuring ocular behavior to evaluate cognitive load. In his report, Beatty concludes that the task-evoked pupillary response fulfills Kahneman’s criteria. Many works support the within-task difference of pupillary responses. For instance, the working memory’s number storage capacity is about seven (Miller, 1956). In this interval, the pupillary diameter is an increasing function of memory load. Once this limit is reached, no further dilation is observed. Beatty (1982) also shows that pupillary responses faithfully reflect variations in processing load between qualitatively different cognitive tasks (memory span, language-processing tasks, complex reasoning, perceptual tasks). Concerning the between-subject variations, Ahern & Beatty (1979) show differences for the digit span task in subjects of high and low psychometrically defined intelligence, and Aminihajibashi et al. (2019) show individual working memory capacity differences in resting-state pupil size. Other works describe differences in pupillary responses for schizophrenia patients in comparison to normal controls (Granholm et al., 1997; Minassian et al., 2004).

Identifying the difficulty level from pupillary responses is challenging because of the between-subject variation. For instance, Fig. 3 illustrates different behavior of the MPDC for different subjects. Tsukahara, Harrison & Engle (2016) found that the baseline pupil size is related to personal cognitive ability. We hypothesize that the best model takes advantage of small personal differences in the baseline. Our approach can predict the difficulty of a task from the pupillary responses that occurred after the stimuli (without the knowledge of the previous responses of the same subject).

## Conclusion

Cognitive load is implicit in all human activities, and as a part of cognitive load, the memorization process is involved in all learning activities.

The main limitation to evaluate the cognitive load from eye-tracking devices is the required sampling frequency. Reading features based on pupil size requires lower frequency than, for instance, detecting eye blinks and fixations. As well, pupil dilation data is accurate and confident in describing the learning activity (Papesh, Goldinger & Hout, 2012).

We are interested in studying the memorization process in real settings, such as learning environments. This implies to solve problems such as distinguishing pupil changes driven by task-related cognitive demands from changes driven by other factors. As Joshi & Gold (2020) suggest, a possible research line is to characterize pupils separately in passive *versus* activate behavioral epochs. For this aim, one can use linear models such as the one proposed in Hoeks & Levelt (1993). In particular, such a strategy must consider intersubject differences of pupil baseline and pupil size variability (Aminihajibashi et al., 2019).

We do believe that other metrics that consider the evolution of the pupil size. For instance, a polygonal representation, can be used beside the proposed ones. We can also develop a learning application to dynamical difficulty adjustment (DDA) based on the flow theory (challenges and skills) (Csikszentmihalyi, 2014). A direct application of this approach can help to avoid boredom or frustration in learners caused by the repetition of a memorization tasks. This can also be an excellent application of reinforcement learning to educational environments.

## Additional Information and Declarations

### Data Availability

The following information was supplied regarding data availability: The code is available at GitHub: https://github.com/HugoMitre/PupillaryResponseClassifier.
